# Evaluating the Impact of Implementing and Scaling-Up the Use of Syphilis Rapid/Point-of-Care Tests: An Interrupted Time Series Analysis of New Syphilis Positivity Rates in Alberta, Canada

**DOI:** 10.1093/cid/ciaf651

**Published:** 2025-12-19

**Authors:** L Alexa Thompson, Jennifer Gratrix, Noel Ives, Kevin Fonseca, Cari Egan, Carla Vetland, Byron M Berenger, Anna Fuezery, A Mark Joffe, Kaitlyn Menard, Laura McDougall, Garret Meyer, Sean B Rourke, Graham Tipples, Stacy Valaire, Allison A Venner, Tom Wong, Hong Yuan Zhou, Ameeta E Singh

**Affiliations:** Population and Public Health, Indigenous Services Canada, Edmonton, Alberta, Canada; STI Services, Primary Care Alberta, Edmonton, Alberta, Canada; Edmonton Remand Centre, Recovery Alberta, Edmonton, Alberta, Canada; Public Health Laboratory, Alberta Precision Laboratories, Calgary, Alberta, Canada; Community Health Sciences, Cummings School of Medicine, University of Calgary, Calgary, Alberta, Canada; Health Systems Knowledge and Evaluation, Acute Care Alberta, Calgary, Alberta, Canada; Public Health Laboratory, Alberta Precision Laboratories, Calgary, Alberta, Canada; Point of Care Testing, Alberta Precision Laboratory, Edmonton, Alberta, Canada; Department of Medicine, University of Alberta, Edmonton, Alberta, Canada; Department of Laboratory Medicine and Pathology, University of Alberta, Edmonton, Alberta, Canada; School of Public Health, University of Alberta, Edmonton, Alberta, Canada; Edmonton STI Clinic, Primary Care Alberta, Edmonton, Alberta, Canada; MAP Centre for Urban Health Solutions, Unity Health Toronto, Toronto, Ontario, Canada; Public Health Laboratory, Alberta Precision Laboratories, Edmonton, Alberta, Canada; STI Services, Primary Care Alberta, Edmonton, Alberta, Canada; Point of Care Testing, Alberta Precision Laboratory, Calgary, Alberta, Canada; Population and Public Health, Indigenous Services Canada, Ottawa, Ontario, Canada; Public Health Laboratory, Alberta Precision Laboratories, Calgary, Alberta, Canada; Department of Medicine, University of Alberta, Edmonton, Alberta, Canada

**Keywords:** syphilis, point-of-care test, rapid tests, interrupted time-series analysis, Canada

## Abstract

**Background:**

In July 2019, a syphilis outbreak was declared in Alberta, Canada, affecting key populations. Syphilis rapid/point-of-care testing (RPOCT) provides opportunities to test individuals in nontraditional settings and provide same-day treatment. This study aimed to evaluate whether RPOCT resulted in a decline in new syphilis positivity rates.

**Methods:**

Starting August 2020, syphilis RPOCTs were implemented in a single (Edmonton) health zone (EDM phase) and in March 2022 were scaled up across the province of Alberta (ProvScaleUp phase). To evaluate the impact of RPOCTs on new syphilis positivity rates, interrupted time-series analyses were used to analyze population-standardized new syphilis positivity rates before, during, and after RPOCT implementation. Generalized linear models assessed percentage declines in new syphilis positivity rates after RPOCT implementation.

**Results:**

In the preintervention period, monthly new syphilis positivity rates significantly increased across Alberta. After RPOCTs were implemented regionally (EDM phase), syphilis positivity rates decreased by an average of 15% (0.25 per 100 000 population). After wider distribution (ProvScaleUp phase), provincial rates decreased by 25% (0.22 per 100 000 population). Rates decreased more in the general versus prenatal population (15.9% vs 12.2%), among males versus females (16.0% vs 14.5%), among those in metropolitan versus urban and rural areas (15.2%, 14.0%, and 12.2%, respectively) and decreased the least among those aged 24–29 (12.5%).

**Conclusions:**

Syphilis RPOCT implementation was associated with a significant decrease in new syphilis positivity rates in a province of a high-income country experiencing a resurgence of heterosexual syphilis among key populations facing barriers to testing and treatment.

## Teaser

The implementation and scale-up of rapid/point-of-care syphilis tests in Alberta, Canada, among underserved populations who face barriers to care, resulted in a notable decline in new syphilis positivity rates.

Syphilis is a resurging bacterial infection transmitted sexually or congenitally, despite being preventable and treatable [1]. The World Health Organization estimated an increase in cases among adults aged 15–49 years by almost 1 million from 2020 to 2022, with 8 million infections in 2022 [2]. Congenital syphilis (CS) can cause fetal loss, stillbirth, preterm birth, low birth weight, developmental delay, blindness, and deafness [3, 4]. In the province of Alberta, Canada, reported cases increased 14-fold between 2014 and 2019, from 161 to 2330 [5]. Congenital cases rose from 0 to 66, with 318 cases between 2019 and 2023 [6]. With rates not seen since the 1940s, the province declared a syphilis outbreak in July 2019 [7].

In Alberta, heterosexual individuals, those with new or multiple partners, and those experiencing houselessness, incarceration, or drug use are disproportionately affected by syphilis [7]. In 2017–2019, 2/3 of women with syphilis in Alberta self-identified as Indigenous, 1/2 were from a neighborhood in the lowest income quintile, 20% reported injection drug use, 10% reported exchange of goods for sex, and 1/3 delivered an infant with CS [8]. Finding and treating infected individuals in traditional settings is made challenging by many factors including geographical barriers and the need to attend follow-up visits [9].

Syphilis rapid/point-of-care testing (RPOCT) enables screening in nontraditional settings and same-day treatment for test-positive individuals. In 2023, in Alberta, the RPOCT for syphilis and HIV study validated 2 dual syphilis/HIV (HIV-1 and HIV-2; referred thereafter as HIV) RPOCT for the diagnosis and treatment of syphilis in a single visit [10]. Eighty-five percent of individuals with infectious syphilis were treated on the same day as their positive RPOCT result. This study aimed to evaluate whether implementation and scale-up of syphilis RPOCTs across the province was associated with decreasing syphilis rates.

## METHODS

### Implementing and Scaling-Up the Use of Syphilis Rapid/Point-of-Care Testings in Alberta

From August 2020 to February 2022, an Edmonton-based RPOCT program (EDM phase) was conducted in 2 emergency departments, a correctional facility and a sexually transmitted infection (STI) clinic in the Edmonton health zone, Alberta (population 1.5 million) [5, 10]. Although the EDM phase program included testing in 1 First Nations community outside of Edmonton zone, the number of tests (n = 60) was small. In March 2022, the dual syphilis/HIV RPOCT was scaled up provincially in provincial health facilities (ProvScaleUp phase) across all 5 Alberta health zones (North, Edmonton, Central, Calgary, and South; population 4.7 million). through the Syphilis and HIV in Acute Care and Community (SHACC) initiative [5, 11]. The RPOCT programs were implemented in geographical areas with high rates of infectious and CS. The clinical laboratory developed and oversaw the implementation to ensure appropriate, high-quality testing support was in place, including supporting quality control/assurance and facilitating RPOCT training for healthcare professionals (registered nurses, licensed practical nurses, and nurse practitioners) Supplementary Table 1 provides additional details about the locations and settings where syphilis RPOCTs were used.

### Syphilis Rapid/Point-of-Care Testing and Patient Management Procedures

Testing for syphilis and HIV was completed according to published methods [10]. Syphilis screening used the reverse sequence algorithm [12]. For those with positive syphilis serology, new infections were staged and treated according to provincial guidelines [7]. Those with positive RPOCT results and no history of syphilis were offered same-visit treatment at the site of testing by providers conducting the testing. Individuals had the option to defer treatment to a follow-up visit. When nurses conducted testing, treatment was provided through patient-specific orders or treatment protocols as authorized by the most responsible provider (MRP). The RPOCT cannot distinguish between new and previous infections; therefore, positive RPOCT syphilis results in persons with previous syphilis were not treated unless history suggested a new infection or exposure. The clinical approach algorithm for results from syphilis RPOCTs is outlined in Supplementary Figure 1.

### Study Design

This retrospective population-based study in Alberta from 1 August 2017 to 31 May 2024 evaluated the impact of RPOCT on new provincial syphilis positivity rates during 3 periods: (1) the preintervention period, when no syphilis RPOCTs were used in the province (1 August 2017–31 July 2020); (2) the EDM phase, when RPOCTS were mostly used in the Edmonton health zone only (1 August 2020–28 February 2022); and (3) the ProvScaleUp phase, during the provincial scale-up of syphilis RPOCTs (1 March 2022–31 May 2024).

### Classification of New Syphilis Diagnoses

All standard syphilis testing in the province is centralized to the Provincial Public Health Laboratory (ProvLab). Testing data over the study period was extracted from the ProvLab Information System (LIS) and included samples with a personal health number (PHN) and conclusive syphilis enzyme immunoassay (EIA) test result. Duplicate accessions, tests canceled for collection or processing errors, and indeterminate, missing, or invalid syphilis EIA results were excluded. Newly confirmed cases were determined by linking PHN with reflex rapid plasma regain (RPR) and *Treponema pallidum* particle agglutination (TPPA) test results and defined as (1) positivity on EIA, RPR, and TPPA tests (new diagnosis/case); (2) positivity on EIA and TPPA tests with nonreactive RPR (new diagnosis/case); or (3) positivity on EIA and RPR with indeterminate TPPA (highly suspected of being a case). For patients with a prior syphilis diagnosis (indicated by previously positive laboratory testing), a new diagnosis was defined as EIA-positive with a 4-fold or higher increase in RPR compared to the last test in the LIS.

### Calculating and Visualizing Syphilis Enzyme Immunoassay Positivity Rates and New Syphilis Positivity Rates

Monthly EIA positivity rates and new syphilis positivity rates were calculated by dividing the monthly number of EIA-positive and new syphilis diagnoses by the number of individuals residing in provincial health zones in each corresponding calendar year and multiplying by 100 000. Residential numbers for denominator calculations were extracted from the Alberta Health Interactive Health Dashboard for Sexually Transmitted Infections [5]. Monthly EIA positivity rates and new syphilis positivity rates were graphed over the monthly volume of EIA tests screened for syphilis over the study period.). The EDM and ProvScaleUp phases are highlighted on each graph. Graphs are stratified by the Edmonton health zone versus provincial rates. Two provincial RPOCT graphs were also visualized: (1) the number of monthly syphilis RPOCTs conducted over the monthly EIA test volume and (2) both syphilis RPOCT and syphilis positivity rates (from standard lab testing) over the monthly EIA test volume. Aggregate raw data for the Edmonton zone and provincial data is included in Supplementary Tables 2 and 3, respectively.

### Interrupted Time-Series Analyses With Generalized Linear Models

An interrupted time-series analysis (ITSA) following segmented linear regression was used to evaluate changes in new syphilis positivity rates during the 3 study phases. The use of an ITSA on Alberta communicable disease data has been described [13]. Briefly, linear regression lines were fit across monthly syphilis positivity rates before, during, and after the specified intervention periods (EDM and ProvScaleUp phases), and the difference in regression lines was compared to determine if there was a significant change in rates. The ITSA was stratified by new syphilis positivity rates in the Edmonton health zone and those across the entire province to evaluate the impacts of regional versus population-wide RPOCT use. Population and testing volume were offset in the statistical analyses to control for variation in annual population numbers and monthly testing volumes. Serial autocorrelation was assessed with the Cumby–Huizinga test. Changes in adjusted rates for the ITSA are reported per 100 000 population. Generalized linear models were also conducted to offset and control for the effects of testing volume and population variation, describe changes in pre- versus postintervention rates as percentages, and evaluate shifts in new syphilis positivity rates across subdemographics (general/prenatal population, age groups, biological sex, health zone, and geographic regions). All statistical analyses were performed using Stata v17.0 (StataCorp, College Station, Texas, USA), and statistical significance was set at α = 0.05.

### Ethics Statement

Ethics approval was provided by the University of Alberta Health Research Ethics Board (Pro00143891). From August 2020 to March 2023, syphilis RPOCT was conducted under an Investigational Testing Authorization (Pro00095828). Patient consent for accessing personally identifying information was waived under the Health Information Act Section 50(1)(a).

## RESULTS

### New Syphilis Diagnoses Over Study Period

**Figure 1.**
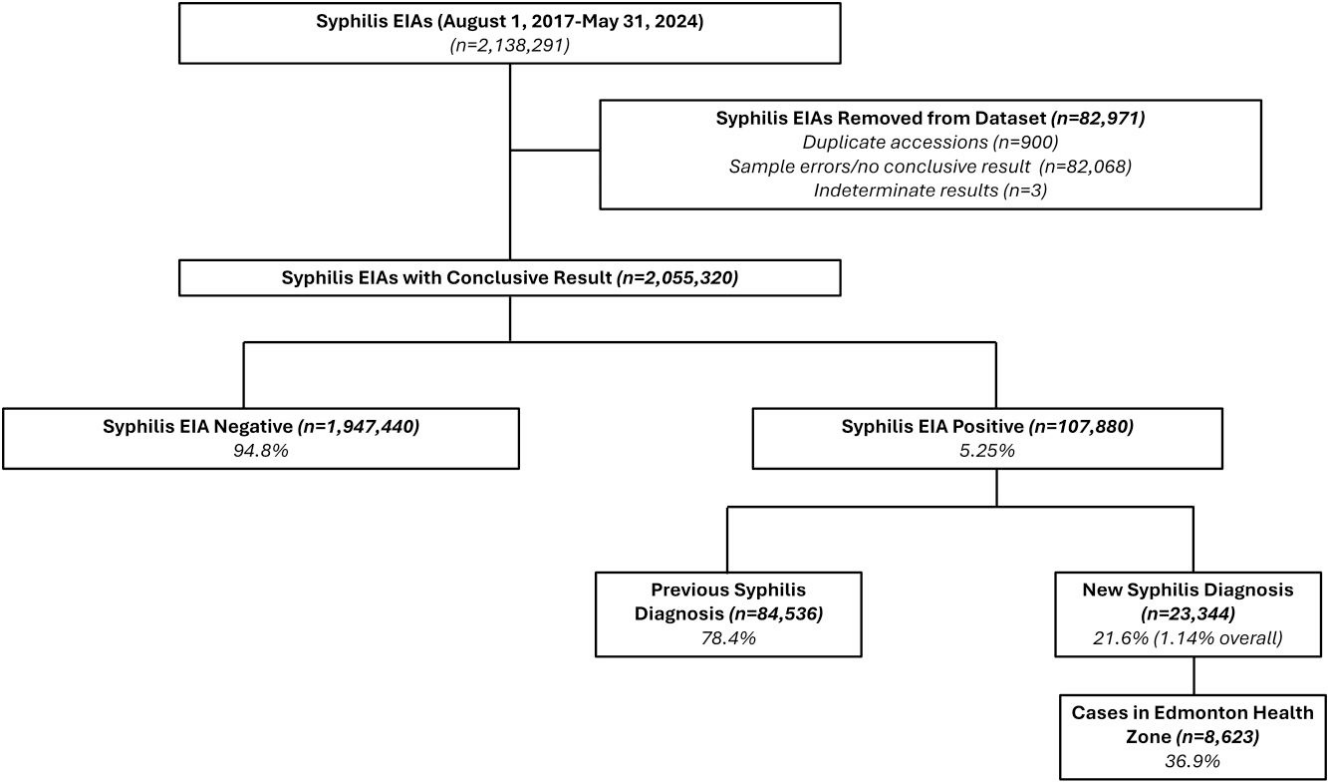
Identification of new syphilis diagnoses across Alberta and in the Edmonton health zone from 1 August 2017–31 May 2024. A new syphilis diagnosis is defined as a confirmed syphilis case based on rapid plasma regain (RPR) and/or *Treponema pallidum* particle agglutination (TPPA) tests. A previous syphilis diagnosis is defined as an individual who is EIA-positive but whose RPR or TPPA tests do not indicate a current active infection. Abbreviation: EIA, enzyme immunoassay.

From 1 August 2017 to 31 May 2024, 2 138 291 EIA tests were conducted in Alberta (Figure 1). Of these, 2 055 320 (96.1%) had a conclusive test result. Of these, 107 880 (5.25%) were positive with 84 536 (78.4%) of tests belonging to people with a previous syphilis diagnosis. Over the study period, there were 23 344 new syphilis diagnoses, equating to 21.6% of those who were syphilis EIA-positive (23 344/107 880) and 1.14% of all syphilis EIAs with a conclusive test result (23 344/2 055 320). Of the 23 344 new diagnoses, 8623 (36.9%) were diagnosed in the Edmonton health zone.

### Trends and Interrupted Time-Series Analysis of Monthly New Syphilis Positivity Rates Across the Edmonton Zone

**Figure 2.**
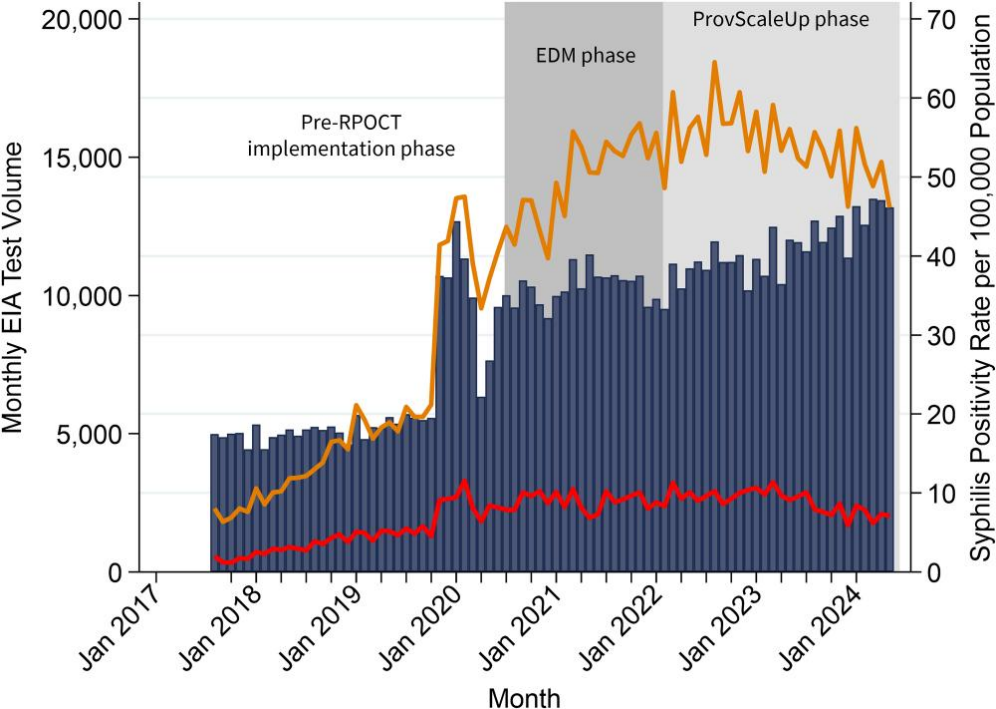
EIA positivity rate (top line) and confirmed new syphilis positivity rate (bottom line) over monthly EIA test volume (bars) in the Edmonton health zone from 1 August 2017 to 31 May 2024, with the Edmonton phase (EDM phase) POCT program running August 2020–February 2022 (dark shading), and the scale-up of syphilis POCT distribution provincially (ProvScaleUp phase) occurring March 2022 onward (light shading). Abbreviations: EIA, enzyme immunoassay; ProvScaleUp, provincial scale-up of syphilis rapid/point of care tests; POCT, point of care test; RPOCT, rapid/point of care test.

Edmonton zone monthly syphilis EIA positivity rates trended upward from August 2017, then peaked in August 2022, and started to decline (Figure 2, orange line). New syphilis positivity rates trended upward from August 2017 to August 2020 (Figure 2, red line) and then leveled off to steady rates until November 2023, where there was a slight decline until May 2024. The monthly EIA test volume trended upward over the study period (Figure 2, blue bars).

In the pre-EDM phase, monthly new syphilis positivity rates in the Edmonton health zone increased by 0.22 per 100 000 population (Table 1, *P* < .001) and stabilized following RPOCT implementation (0.89 per 100 000 population, *P* = .272) and in the follow-up period (−0.02 per 100 000 population, *P* = .616). However, new syphilis positivity rates decreased by 0.25 per 100 000 population post-EDM versus pre-EDM phase (*P* < .001). After the ProvScaleUp phase, monthly new syphilis positivity rates decreased across the Edmonton zone by 0.09 per 100 000 population (*P* = .046), similar to the decrease in new syphilis positivity rates seen after the EDM phase was implemented (−0.06 per 100 000 population, *P* = .352). The post-trend figure for new syphilis positivity rates in the Edmonton zone is included in Supplementary Figure 2 (left).

**Table 1.** Interrupted Time-Series Analysis for Monthly New Syphilis Positivity Rates (per 100 000 Population) Across Edmonton Health Zone From 1 August 2017 to 31 May 2024, Stratified by the Edmonton Phase (EDM Phase) POCT Program (August 2020–February 2022) and the Scale-Up of Syphilis POCT Distribution Provincially (ProvScaleUp Phase; March 2022 Onward)

EDM Phase POCT Program (August 2020–February 2022)	Baseline Average Monthly Rate Before EDM Phase	Change in Average Monthly Rates Before EDM Phase Was Implemented (August 2017–August 2020)		Change in Average Monthly Rate When EDM Phase Was Implemented (August 2020)		Change in Average Monthly Rates in Follow-Up Period After EDM Phase Was Implemented (September 2020–May 2024)		Change In Average Monthly Rates After EDM Phase Was Implemented (August 2020–May 2024), Relative to Before EDM Phase (August 2017–August 2020)	
	β estimate (per 100 000)	β estimate (per 100 000)	*P* value	β estimate (per 100 000)	*P* value	β estimate (per 100 000)	*P* value	β estimate (per 100 000)	*P* value
	1.03	0.22	<.001*	0.89	.272	−0.02	.616	−0.25	<.001*
ProvScaleUp Phase POCT Program (March 2022 Onward)	Baseline average monthly rate before ProvScaleUp	Change in average monthly rates before ProvScaleUp was implemented (August 2017–March 2022)		Change in average monthly rate when ProvScaleUp was implemented (March 2022)		Change in average monthly rates in follow-up period after ProvScaleUp was implemented (March 2022–May 2024)		Change in average monthly rates after ProvScaleUp was implemented (March 2022–May 2024), relative to the EDM phase (August 2020–March 2022)	
	β estimate (per 100 000)	β estimate (per 100 000)	*P* value	β estimate (per 100 000)	*P* value	β estimate (per 100 000)	*P* value	β estimate (per 100 000)	*P* value
	1.73	0.18	<.001*	0.87	.279	−0.09	.046*	−0.06	.352

β estimates for average monthly syphilis positivity rates are standardized and reported per 100 000 population.

Abbreviation: POCT, point-of-care testing.

^*^ *P* < .05 is significant.

Across the Edmonton zone, syphilis positivity rates declined by 25.0% post- versus pre-EDM phase (Table 2). Rates decreased more across the general versus prenatal population (26.2% vs 15.9%), among males versus females (27.5% vs 23.4%), and decreased the least among those aged 24–29 (20.6%) and 30–34 (22.7%).

**Table 2.** Decline in Syphilis Rates Across Edmonton Health Zone After Commencing the Edmonton Phase (EDM Phase) POCT Program in August 2020, Stratified by Demographics

	IRR (95% CI)	% Decline (Post-EDM Phase vs Pre-EDM Phase)	*P* Value
Population			
Total population	0.750 (.749–.751)	25.0%	<.001*
General population	0.738 (.737–.739)	26.2%	<.001*
Prenatal population	0.841 (.840–.842)	15.9%	<.001*
Age group			
0–14	0.740 (.739–.741)	26.0%	<.001*
15–19	0.712 (.711–.713)	28.8%	<.001*
20–24	0.742 (.741–.743)	25.8%	<.001*
24–29	0.794 (.793–.795)	20.6%	<.001*
30–34	0.773 (.772–.774)	22.7%	<.001*
35–39	0.729 (.728–.730)	27.1%	<.001*
40–49	0.686 (.685–.687)	31.4%	<.001*
50–59	0.743 (.742–.744)	25.7%	<.001*
60+	0.672 (.671–.673)	32.8%	<.001*
Biological sex			
Male	0.725 (.724–.726)	27.5%	<.001*
Female	0.766 (.765–.767)	23.4%	<.001*

Abbreviations: IRR, incident rate ratio; POCT, point-of-care testing; 95% CI, 95% confidence interval.

^*^ *P* < .05 is significant using generalized linear models.

### Trends and Interrupted Time-Series Analysis of Monthly New Syphilis Positivity Rates Across Alberta

**Figure 3.**
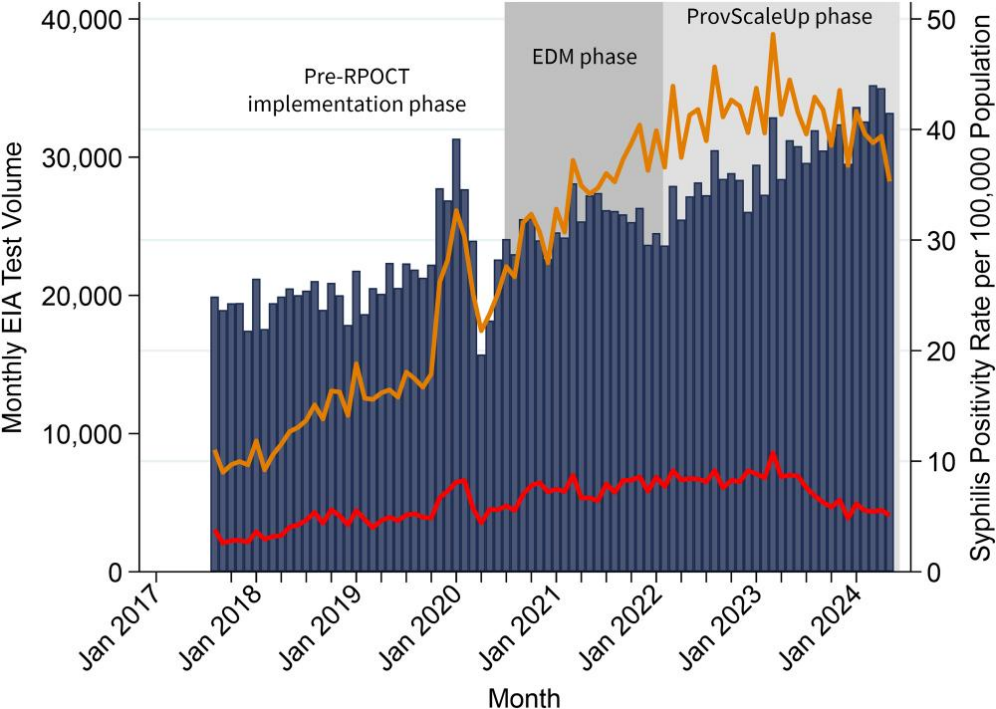
EIA positivity rate (top line) and confirmed new syphilis positivity rate (bottom line) over monthly EIA test volume (bars) in Alberta from 1 August 2017 to 31 May 2024, with the Edmonton phase (EDM phase) POCT program running August 2020–February 2022 (dark shading), and the scale-up of syphilis POCT distribution provincially (ProvScaleUp phase) occurring March 2022 onward (light shading). Abbreviations: EIA, enzyme immunoassay; POCT, point of care test; ProvScaleUp, provincial scale-up of syphilis rapid/point of care tests; RPOCT, rapid/point of care test.

Provincially, monthly syphilis EIA positivity rates rose, peaking in March 2023 (Figure 3, orange line). New syphilis positivity rates followed the same trend (Figure 3, red line). Monthly EIA test volume trended upward over the study period (Figure 3, blue bars), except for a drop in testing volume in April 2020 attributed to the coronavirus-2019 (COVID-19) pandemic. The monthly volume of syphilis RPOCTs conducted provincially over the study is shown in Supplementary Figure 3, and the syphilis POCT positivity rate versus overall syphilis positivity rate is shown in Supplementary Figure 4.

Pre-ProvScaleUp phase, monthly new syphilis positivity rates increased by 0.10 per 100 000 population (Table 3, *P* < .001). New syphilis positivity rates decreased by 0.12 per 100 000 population post-ProvScaleUp phase (*P* = .004) and were significantly lower compared to rates observed during the EDM phase (−0.22 per 100 000 population, *P* < .001).

**Table 3.** Interrupted Time-Series Analysis for Monthly New Syphilis Positivity Rates (per 100 000 Population) Across Alberta From 1 August 2017 to 31 May 2024, Stratified by the Edmonton Phase (EDM Phase) POCT Program (August 2020–February 2022) and the Scale-Up of Syphilis POCT Distribution Provincially (ProvScaleUp Phase; March 2022 Onward)

EDM Phase POCT Program (August 2020–February 2022)	Baseline Average Monthly Rate Before EDM Phase	Change in Average Monthly Rates Before EDM Phase Was Implemented (August 2017–August 2020)		Change in Average Monthly Rate When EDM Phase Was Implemented (August 2020)		Change in Average Monthly Rates in Follow-Up Period After EDM Phase Was Implemented (September 2020–May 2024)		Change In Average Monthly Rates After EDM Phase was Implemented (August 2020-May 2024), Relative to Before EDM Phase (August 2017-August 2020)	
	β estimate (per 100 000)	β estimate (per 100 000)	*P* value	β estimate (per 100 000)	*P* value	β estimate (per 100 000)	*P* value	β estimate (per 100 000)	*P* value
	2.92	0.11	<.001*	−0.08	.889	0.10	.004*	−0.01	.761
ProvScaleUp Phase POCT Program (March 2022 Onward)	Baseline average monthly rate before ProvScaleUp	Change in average monthly rates before ProvScaleUp was implemented (August 2017–March 2022)		Change in average monthly rate when ProvScaleUp was implemented (March 2022)		Change in average monthly rates in follow-up period after ProvScaleUp was implemented (March 2022–May 2024)		Change in average monthly rates after ProvScaleUp was implemented (March 2022–May 2024), relative to the EDM phase (August 2020–March 2022)	
	β estimate (per 100 000)	β estimate (per 100 000)	*P* Value	β estimate (per 100 000)	*P* Value	β estimate (per 100 000)	*P* Value	β estimate (per 100 000)	*P* Value
	3.00	0.10	<.001*	0.87	.171	−0.12	.004*	−0.22	<.001*

β estimates for average monthly syphilis positivity rates are standardized and reported per 100 000 population.

Abbreviation: POCT, point-of-care testing.

^*^ *P* < .05 is significant.

There were no declines in provincial new syphilis positivity rates when RPOCTs were limited to the EDM phase (−0.08 per 100 000 population, *P* = .889 when the EDM phase was implemented, 0.10 per 100 000 population, *P* = .004 post-EDM phase, and −0.01 per 100 000 population, *P* = .761 post-EDM phase relative to the pre-EDM phase). The post-trend figure for syphilis positivity rates across Alberta is included in Supplementary Figure 2 (right). Provincial positivity rates declined by 15.0% after the ProvScaleUp phase (Table 4). Rates decreased more across the general versus prenatal population (15.9% vs 12.2%), among males versus females (16.0% vs 14.5%), across the Edmonton and Calgary health zones (15.9% for both), and among those in metropolitan versus urban versus rural regions (15.2%, 14.0%, and 12.2%, respectively) and decreased the least among those aged 24–29 (12.5%).

**Table 4.** Decline in Syphilis Rates Across Alberta After Commencing the Provincial Scale-Up (ProvScaleUp Phase) of POCT in March 2022, Stratified by Demographics

	IRR (95% CI)	% Decline (Post-ProvScaleUp Phase vs Pre-ProvScaleUp Phase)	*P* Value
Population			
Total population	0.850 (.849–.851)	15.0%	<.001*
General population	0.841 (.840–.842)	15.9%	<.001*
Prenatal population	0.878 (.877–.879)	12.2%	<.001*
Age group			
0–14	0.859 (.858–.860)	14.1%	<.001*
15–19	0.810 (.809–.811)	19.0%	<.001*
20–24	0.846 (.845–.847)	15.4%	<.001*
24–29	0.875 (.874–.876)	12.5%	<.001*
30–34	0.858 (.857–.859)	14.2%	<.001*
35–39	0.822 (.821–.823)	17.8%	<.001*
40–49	0.793 (.792–.794)	20.7%	<.001*
50–59	0.866 (.865–.867)	13.4%	<.001*
60+	0.764 (.763–.765)	23.6%	<.001*
Biological sex			
Male	0.840 (.839–.841)	16.0%	<.001*
Female	0.855 (.854–.856)	14.5%	<.001*
Health zone			
North	0.910 (.909–.911)	9.00%	<.001*
Edmonton	0.841 (.840–.842)	15.9%	<.001*
Central	0.908 (.907–.909)	9.20%	<.001*
Calgary	0.841 (.840–.842)	15.9%	<.001*
South	0.910 (.909–.911)	9.00%	<.001*
Geographic region			
Metropolitan	0.848 (.847–.849)	15.2%	<.001*
Urban	0.860 (.859–.861)	14.0%	<.001*
Rural	0.878 (.877–.879)	12.2%	<.001*

Abbreviations: IRR, incident rate ratio; POCT, point-of-care testing; 95% CI, 95% confidence interval.

^*^ *P* < .05 is significant using generalized linear models.

## DISCUSSION

Our study, conducted over nearly 7 years in a western Canadian province, found that implementing RPOCTs in a single health zone (Edmonton) led to a 25% (0.25 per 100 000 population) decrease in new syphilis positivity rates in that zone. After wider distribution (ProvScaleUp phase), provincial rates also decreased by 15% (0.22 per 100 000 population). Although 36.9% of all new syphilis cases were diagnosed in a single health zone (Edmonton) during the study period, restriction of RPOCT to that zone was insufficient to have an impact on provincial positivity rates. Provincial rates did not decline until RPOCTs were implemented throughout all 5 health zones.

Our study is the first to evaluate the impact of a systematic scale-up of syphilis RPOCT on new syphilis positivity rates in a high-income country. Our findings align with a recent modeling study from Canadian Arctic communities, which estimated that deploying syphilis rapid diagnostic tests over 5 and 10 years could avert 33% and 37% of new syphilis infections, respectively [14]. Following the compelling findings of an RPOCT validation study in Alberta [10], the neighboring province of Saskatchewan implemented RPOCT in March 2022. A Canadian Institute for Health Research initiative called *Ayaangwaamiziwin*, an Ojibway word meaning “carefulness and preparedness,” will continue to scale up syphilis/HIV RPOCT over the next 3–5 years in Alberta, Saskatchewan, and Manitoba, 3 prairie provinces experiencing high rates of infectious syphilis and HIV in similar key populations [15]. Interestingly, Saskatchewan and Manitoba have also observed drops in syphilis rates in recent years [16], further suggesting a possible association between the initial use and scale-up of syphilis RPOCTs and syphilis rates declining. The Syphilis Rapid POCT and Immediate Treatment Evaluation (SPRITE) study, implemented in June 2023, in Ontario, Canada, has also seen a recent decline in syphilis rates [17].

We noted fewer declines in the prenatal population, which is concerning given the significant rise in CS cases seen across Canada over the past 2 decades [18]. Additionally, those aged 24–29 and 30–34 years saw fewer declines in new syphilis positivity rates, yet represent 2 of the age groups most affected by syphilis in Alberta and Canada [6, 16]. These findings collectively highlight the need to couple syphilis RPOCT with programs that can reach affected populations. Our experience has also highlighted the need for a holistic approach to address social and structural barriers including racism, stigma and discrimination, substance use, houselessness, and food insecurity that prevent individuals from accessing STBBI testing and treatment [19, 20]. Engaging community-based organizations and members of key populations is essential in developing culturally appropriate and safe strategies [21]. Providing opportunistic syphilis screening for individuals in emergency departments (EDs), including pregnant persons at risk for syphilis, has been shown to reach those who may only access care through EDs for nonscreening reasons [10, 22]. Offering screening as part of other targeted programs, such as HIV care and prevention services and viral hepatitis services, may be helpful in reaching populations with overlapping risk factors for STBBI [23]. Additionally, screening in nontraditional settings, such as corrections and harm reduction programs, and the use of incentives for testing and treatment may also be necessary [10, 11, 24, 25].

### Limitations

The retrospective, observational nature of the study limits our ability to establish a causative link between syphilis RPOCTs and declining rates. Other factors, such as partial host immunity to syphilis and oscillations in disease incidence, may have contributed to the decline [26, 27]. Indeed, a study from Alberta reported cycling of disease rates in approximately 10-year cycles over a 44-year time period [28]. However, our observation that rates fell in the Edmonton zone after implementing the EDM phase but not in other health zones (outlined in Supplementary Table 4) suggests otherwise. It is also noteworthy that the decline in rates occurred within 3–4 years of RPOCT implementation, a steeper decline than would be expected to naturally occur over an 8–11-year cycle [26].

Another possible explanation for declining rates may have been the implementation of additional interventions, but since the EDM phase took place during the COVID-19 pandemic, other services were largely discontinued.

Our findings are also limited by only having access to aggregate provincial numbers of RPOCTs recorded in the LIS and our inability to include all RPOCT conducted in Alberta. It is estimated that 2000–2500 other tests were funded by Indigenous Services Canada and used by organizations working with Indigenous persons (personal communication, Dr Lauren Bilinsky, Indigenous Services Canada, Alberta region). Therefore, our RPOCTs and RPOCT positivity rates are likely underestimates of the true number of RPOCTs conducted.

We did not focus on treatment outcomes in this study as other Alberta-based studies have reported on this with RPOCT use [10, 11], but it is presumed that RPOCT use was associated with declining new syphilis positivity rates as the treatment of individuals at the same visit as a positive RPOCT would limit ongoing transmission to others.

Lastly, the study included individuals who had standard lab testing for syphilis [29], but excluded individuals without a recorded PHN or with an inconclusive result. People previously diagnosed in provinces outside of Alberta may also be represented as new cases in our dataset. These limitations are offset by the fact that the data represents population-based testing across Alberta, so the exclusion of this small number of individuals is unlikely to significantly alter our findings. Testing volumes, changes in provincial populations, and potential confounders were adjusted for in the generalized linear models as much as possible, further supporting this idea. Although 82 068 line lists of information were removed from the initial dataset due to an inconclusive syphilis EIA result, 81 364 (99.1%) of these had a conclusive syphilis EIA test result recorded in a separate line list in the final dataset analyzed.

## CONCLUSIONS

Scaled implementation of syphilis RPOCTs resulted in a significant decrease in syphilis positivity rates. Syphilis RPOCTs should be considered an important public health tool to combat outbreaks and improve testing and treatment initiatives. More work is needed in Alberta to target important at-risk subgroups who saw fewer declines in rates.

## Supplementary Material

ciaf651_Supplementary_Data

## Supplementary Data

Supplementary materials are available at *Clinical Infectious Diseases* online. Consisting of data provided by the authors to benefit the reader, the posted materials are not copyedited and are the sole responsibility of the authors, so questions or comments should be addressed to the corresponding author.
