# Supplementary material for: Evaluating the Impact of Implementing and Scaling-Up the Use of Syphilis Rapid/Point-of-Care Tests: An Interrupted Time Series Analysis of New Syphilis Positivity Rates in Alberta, Canada
Source: Clin Infect Dis. 2025 Dec 19;82(4):e826–35. doi: 10.1093/cid/ciaf651 (PMC13131939; doi:10.1093/cid/ciaf651)
Supplement: ciaf651_Supplementary_Data [file ciaf651_supplementary_data.zip › ITSA AB_Manuscript_Supplementary Materials_FINAL_02Nov2025.docx]

**Supplementary Materials**

Table of contents:

**Supplementary Figure 1. Algorithm for clinical approach to results from syphilis rapid point-of-care tests (RPOCTs).**

**Supplementary Figure 2. Interrupted time-series analysis post-trend figures for monthly new syphilis positivity rates across Edmonton health zone (left) and Alberta (right), showing average monthly new syphilis positivity rates before any POCT interventions (August 2017-July 2020; coded 691-726), during the Edmonton phase (EDM phase) POCT program in the Edmonton health zone (August 2020-February 2022; coded 727-745 with intervention starting at 727), and during the provincial scale-up (ProvScaleUp phase) of the syphilis POCT program (March 2022 onwards; coded 746-772 with intervention starting at 746).**

**Supplementary Figure 3**. **Number of syphilis POCTs performed (orange) over monthly EIA test volume (blue bars) in Alberta from August 1, 2017-May 31, 2024, with the Edmonton phase (EDM phase) POCT program running August 2020-February 2022 (dark grey shading), and the scale-up of syphilis POCT distribution provincially (ProvScaleUp phase) occurring March 2022 onwards (light grey shading).**

**Supplementary Figure 4. Confirmed new syphilis positivity rate (orange) and syphilis POCT positivity rate (red) over monthly EIA test volume (blue bars) in Alberta from August 1, 2017-May 31, 2024, with the Edmonton phase (EDM phase) POCT program running August 2020-February 2022 (dark grey shading), and the scale-up of syphilis POCT distribution provincially (ProvScaleUp phase) occurring March 2022 onwards (light grey shading).**

**Supplementary Table 1. Details on the implementation and provincial scale-up of syphilis rapid point-of-care tests (RPOCTs) in Alberta.**

**Supplementary Table 2. Aggregate raw data of syphilis testing numbers and positivity rates in the Edmonton zone by month and year over the study period (August 1, 2017-May 31, 2024).**

**Supplementary Table 3. Aggregate raw data of syphilis testing numbers and positivity rates across the province by month and year over the study period (August 1, 2017-May 31, 2024).**

**Supplementary Table 4. Alberta Health reported infectious syphilis rates per 100,000 population from 2017-2023, stratified by health zone.**

**
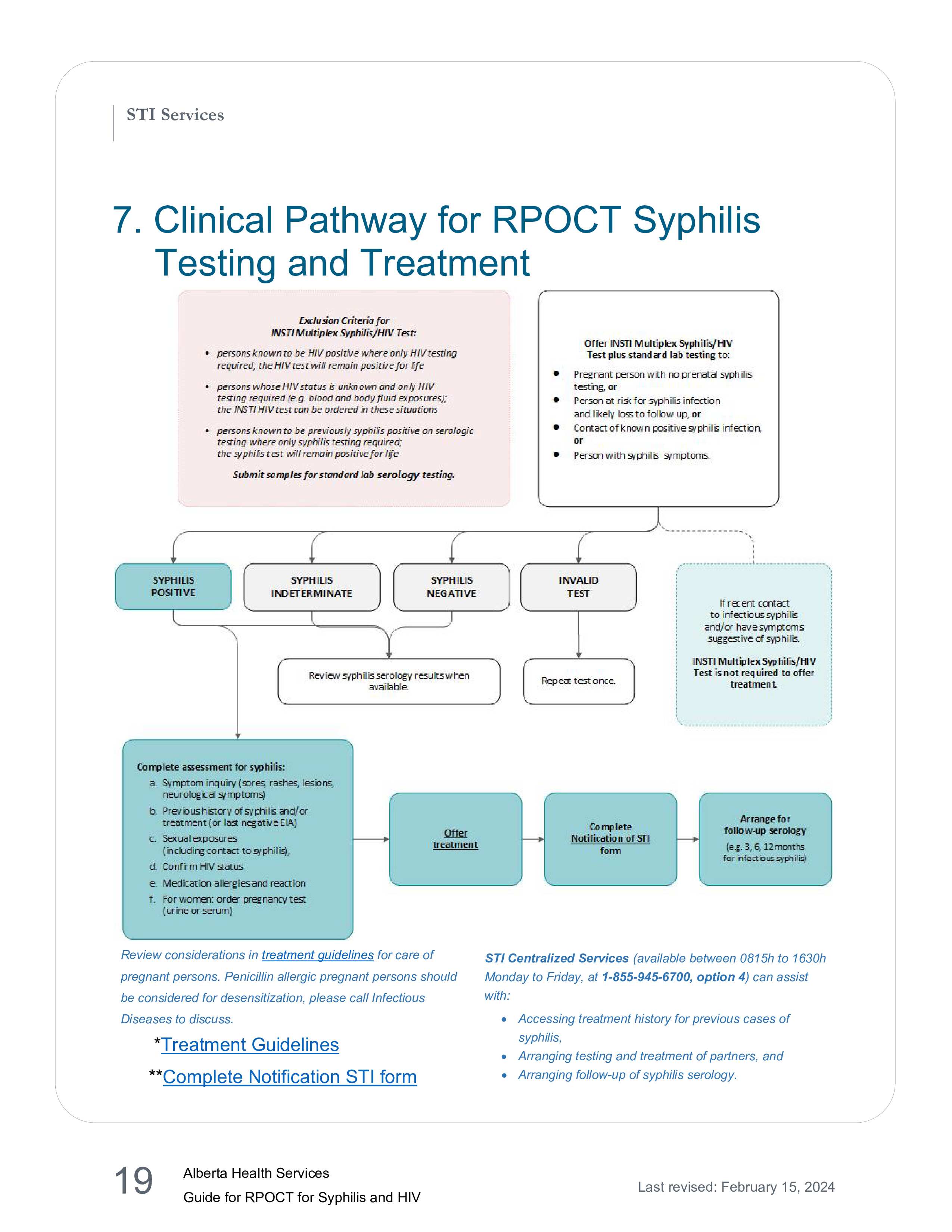
**

**Supplementary Figure 1. Algorithm for clinical approach to results from syphilis rapid point-of-care tests (RPOCTs).**


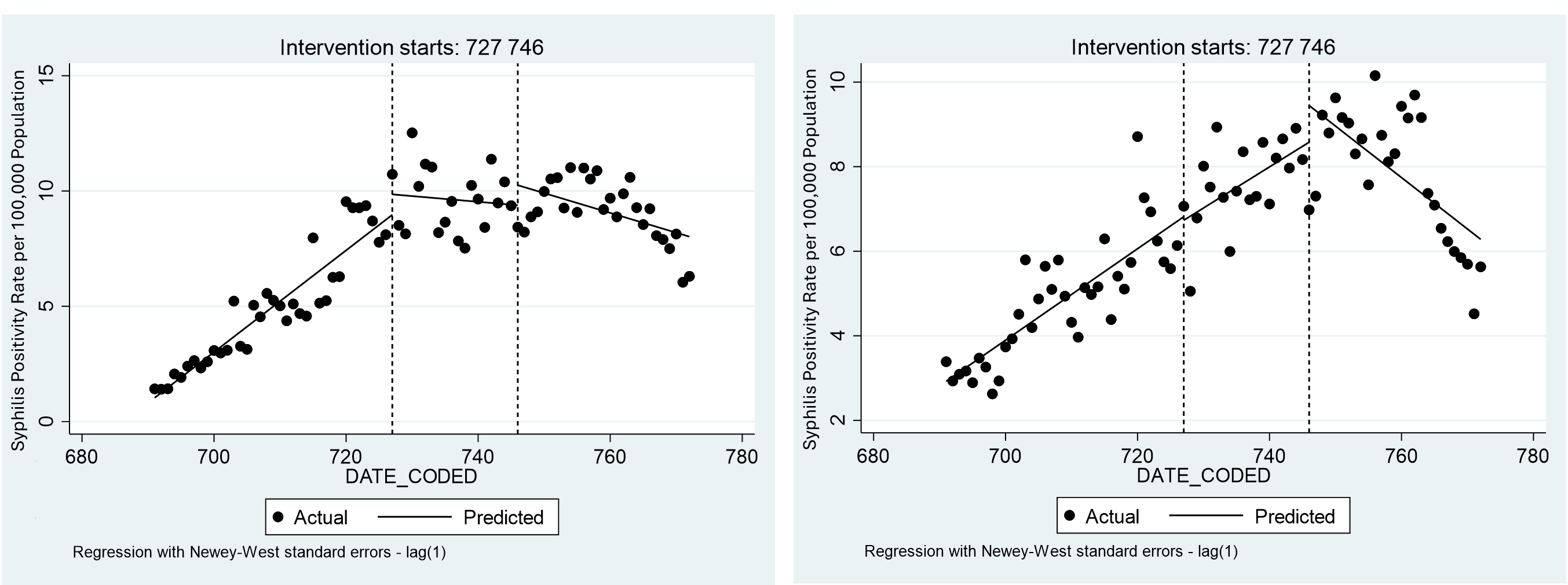


**Supplementary Figure 2. Interrupted time-series analysis post-trend figures for monthly new syphilis positivity rates across Edmonton health zone (left) and Alberta (right), showing average monthly new syphilis positivity rates before any POCT interventions (August 2017-July 2020; coded 691-726), during the Edmonton phase (EDM phase) POCT program in the Edmonton health zone (August 2020-February 2022; coded 727-745 with intervention starting at 727), and during the provincial scale-up (ProvScaleUp phase) of the syphilis POCT program (March 2022 onwards; coded 746-772 with intervention starting at 746).**


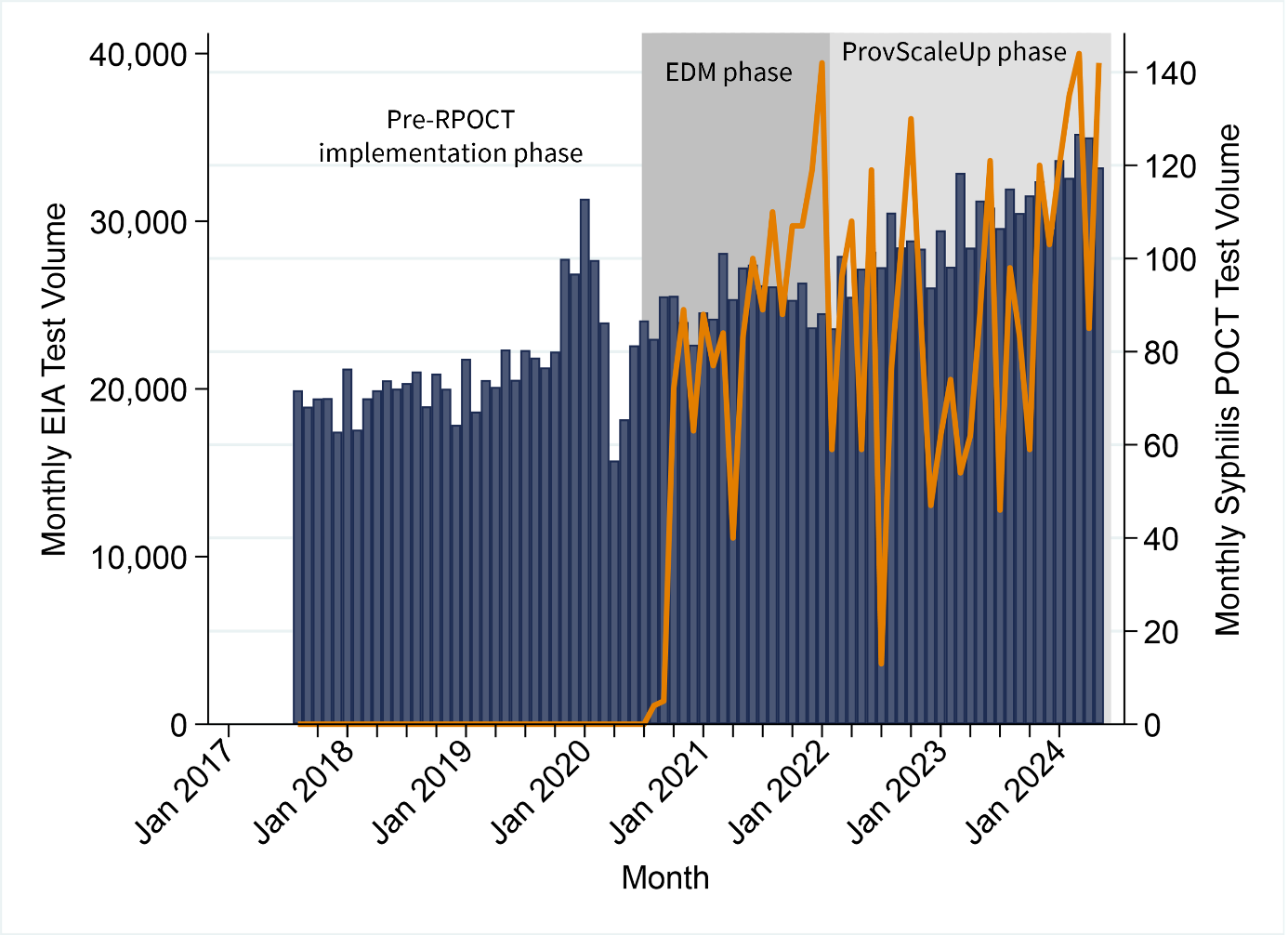


**Supplementary Figure 3**. **Number of syphilis POCTs performed (orange) over monthly EIA test volume (blue bars) in Alberta from August 1, 2017-May 31, 2024, with the Edmonton phase (EDM phase) POCT program running August 2020-February 2022 (dark grey shading), and the scale-up of syphilis POCT distribution provincially (ProvScaleUp phase) occurring March 2022 onwards (light grey shading).** EIA; enzyme immunoassay. POCT; point-of-care-test.


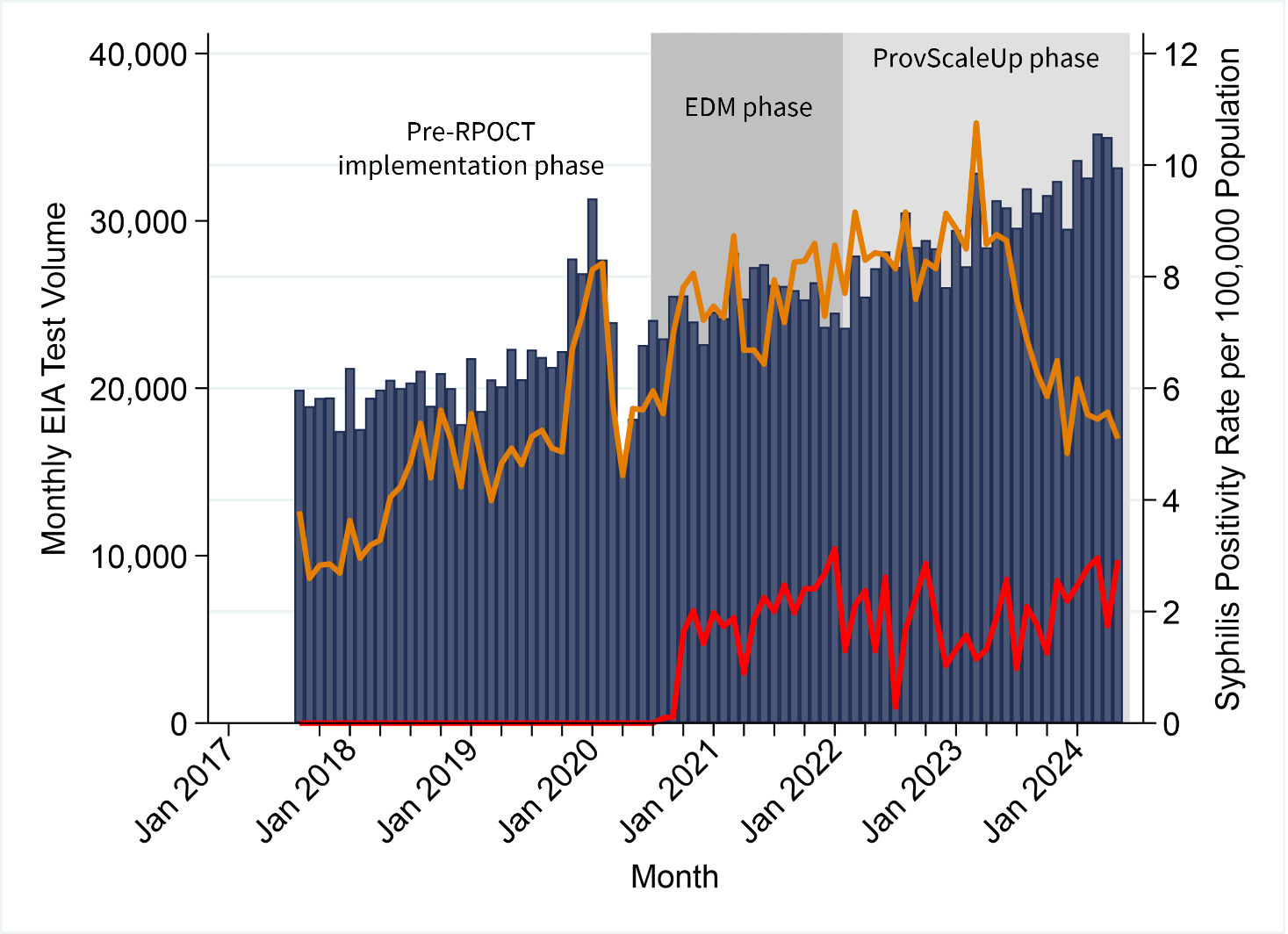


**Supplementary Figure 4. Confirmed new syphilis positivity rate (orange) and syphilis POCT positivity rate (red) over monthly EIA test volume (blue bars) in Alberta from August 1, 2017-May 31, 2024, with the Edmonton phase (EDM phase) POCT program running August 2020-February 2022 (dark grey shading), and the scale-up of syphilis POCT distribution provincially (ProvScaleUp phase) occurring March 2022 onwards (light grey shading).** EIA; enzyme immunoassay. POCT; point-of-care-test.

| **Supplementary Table 1. Details on the implementation and provincial scale-up of syphilis rapid point-of-care tests (RPOCTs) in Alberta.** | | |
| --- | --- | --- |
| *Location* | *Date (Month, Year)* | *Health Zone* |
| Edmonton STI Clinic | March 2020 | Edmonton |
| Edmonton PNN Office | March 2020 | Edmonton |
| Royal Alexandra Hospital | October 2023 | Edmonton |
| Misericordia Hospital | December 2023 | Edmonton |
| Grey Nuns Hospital | February 2024 | Edmonton |
| Fort McMurray STI Clinic | September 2022 | North |
| Grande Prairie Regional Hospital | November 2023 | North |
| North Zone PNN Office | December 2023 | North |
| Wetaskiwin PNN Office | June 2022 | Central |
| Rest of Central Zone PNN Offices | September 2022 | Central |
| Wetaskiwin Hospital | April 2024 | Central |
| Red Deer Hospital | April 2024 | Central |
| Calgary STI Clinic | May 2023 | Calgary |
| Calgary Zone PNN Office | December 2023 | Calgary |
| Peter Lougheed Hospital | June 2024 | Calgary |
| South Zone PNN Office | May 2023 | South |
| Cardston Clinic | July 2024 | South |
| Pincher Creek Medical Clinic | July 2024 | South |
| PNN: partner notification nurse. STI: sexually transmitted infection. | | |

**Supplementary Table 2. Aggregate raw data of syphilis testing numbers and positivity rates in the Edmonton zone by month and year over the study period (August 1, 2017-May 31, 2024).**

Please see separate CSV. attachment “Supplementary Table 2_CID MS CID-NPOCD-009 Raw Data Edmonton Zone” to access the aggregate raw data for the Edmonton zone analyses.

**Supplementary Table 3. Aggregate raw data of syphilis testing numbers and positivity rates across the province by month and year over the study period (August 1, 2017-May 31, 2024).**

Please see separate CSV. attachment “Supplementary Table 3_CID MS CID-NPOCD-009 Raw Data Provincial Zones” to access the aggregate raw data for the province-wide analyses.

| **Supplementary Table 4. Alberta Health reported infectious syphilis rates per 100,000 population from 2017-2023, stratified by health zone.** | | | | | |
| --- | --- | --- | --- | --- | --- |
|  | **South Zone** | **Calgary Zone** | **Central Zone** | **Edmonton Zone** | **North Zone** |
| **2017** | 3.63 | 12.2 | 8.04 | 16.7 | 13.1 |
| **2018** | 8.51 | 13.2 | 23.4 | 66.6 | 57.4 |
| **2019** | 10.4 | 12.4 | 39.4 | 95.8 | 111.5 |
| **2020** | 14.5 | 22.6 | 54.5 | 80.3 | 139.2 |
| **2021** | 35.6 | 38.6 | 82.1 | 90.3 | 162.2 |
| **2022** | 59.2 | 43.7 | 88.3 | 78.5 | 161.6 |
| **2023** | 58.6 | 33.7 | 51.9 | 64.4 | 132.4 |
| Data extracted from the Interactive Health Data Application dashboard. | | | | | |
